# Pheochromocytoma: A Troublesome Tumor

**DOI:** 10.7759/cureus.45490

**Published:** 2023-09-18

**Authors:** Tirath Patel, Leah A Singleton, Michael Mejia, Amanda Senior, Richard M Millis

**Affiliations:** 1 Department of Pathophysiology, American University of Antigua, St. John’s, ATG

**Keywords:** von hippel-lindau disease, multiple endocrine neoplasia, differential diagnosis, abdominal radiology, adrenal pheochromocytoma

## Abstract

This report presents the case of a 45-year-old man with a history of episodic headaches, palpitations, and sweating for the past six months. His blood pressure on admission was 170/100 mmHg. The patient was diagnosed with pheochromocytoma confirmed by elevated levels of plasma catecholamines and metanephrines. CT imaging revealed a 3 cm mass in the left adrenal gland with evidence of local invasion into the surrounding tissues. The patient underwent a laparoscopic adrenalectomy and was discharged on the third postoperative day with normal blood pressure. Histopathological examination confirmed the diagnosis of pheochromocytoma. The patient was followed for six months postoperatively with the resolution of symptoms and no evidence of tumor recurrence on imaging. Recurrence involves complex environment-gene interactions that are poorly understood. The diagnosis of pheochromocytoma could take several weeks to several years mainly because the symptoms are nonspecific and episodic. Although sudden death is rare, the debilitations associated with pheochromocytoma are often multisystemic with cardiovascular, emotional, and metabolic components. This case report highlights the importance of early diagnosis, appropriate management, and follow-up for pheochromocytoma.

## Introduction

Pheochromocytoma is a rare tumor that develops in the adrenal medulla's chromaffin cells. It causes an unregulated and excessive production of the hormones epinephrine and norepinephrine [1]. It accounts for less than 0.2% of all diagnosed hypertension cases [2]. This tumor can occur sporadically or as a part of an inherited syndrome like multiple endocrine neoplasia (MEN) or von Hippel-Lindau (VHL) disease [3]. Pheochromocytoma can present with a wide range of symptoms including episodic headaches, sweating, palpitations, anxiety, and hypertension [4]. The diagnosis is challenging because of the multisystemic, nonspecific, and episodic nature of the symptoms, and hence, imaging and biochemical testing play a crucial role. Surgical resection is the mainstay of treatment.

## Case presentation

**Figure 1:**
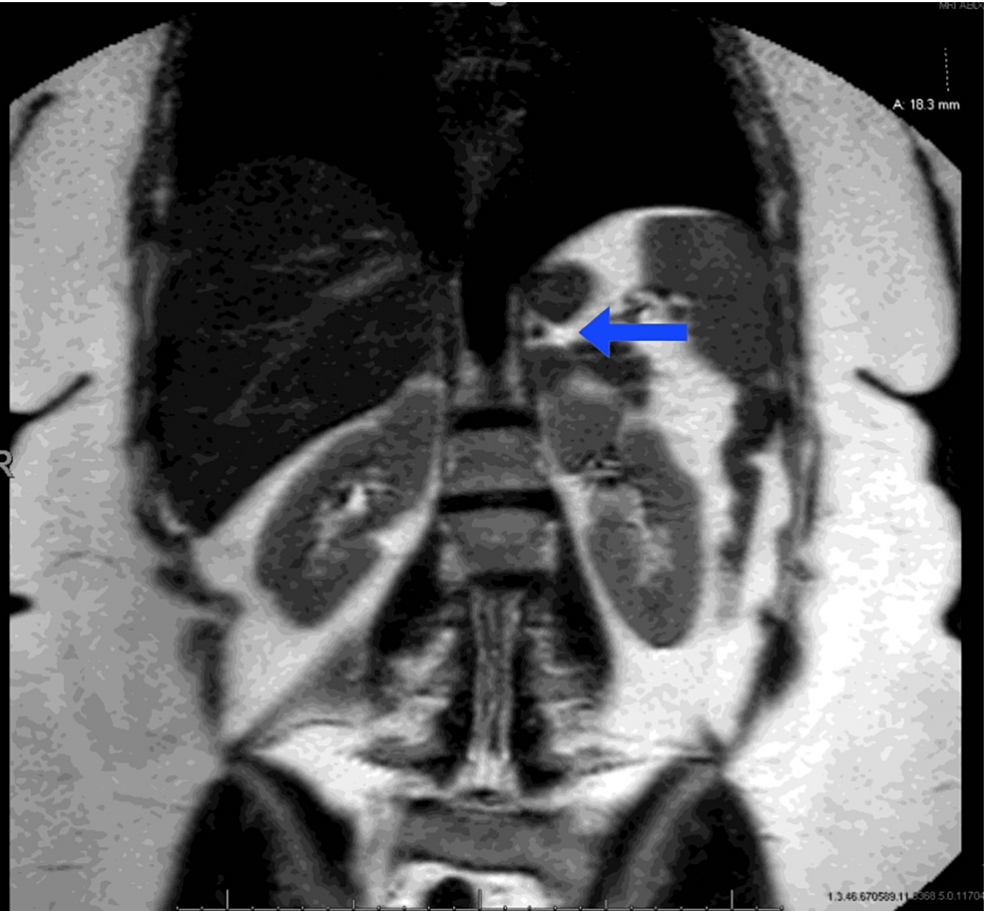
Abdominal magnetic resonance imaging (MRI) scan. Arrow points to a clearly visible 3 cm pheochromocytoma in the left retroperitoneal flank region of a 45-year-old man who presented with a history of episodic headaches, palpitations, and sweating for the past six months.

**Figure 2:**
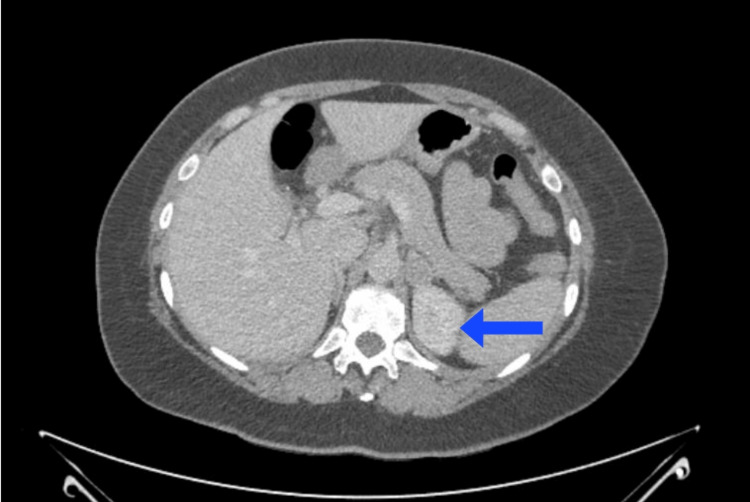
Abdominal computed tomography (CT) scan. Arrow points to a 3 cm pheochromocytoma deep in the left retroperitoneal flank region of a 45-year-old man who presented with a history of episodic headaches, palpitations, and sweating for the past six months.

A 45-year-old man presented with a history of episodic headaches, palpitations, and sweating for the past six months. He also reported feeling anxious and irritable during these episodes. He denied any history of hypertension or other chronic medical conditions. There was no family history of pheochromocytoma or other endocrine disorders. Physical examination was unremarkable except for an elevated blood pressure of 170/100 mmHg (normal < 120/80 mmHg). Laboratory investigations revealed elevated levels of plasma catecholamines (epinephrine: 550 pg/mL (normal < 100 pg/mL), norepinephrine: 700 pg/mL (normal 100-450 pg/mL), and metanephrines (metanephrine: 1800 pg/mL (normal <165 pg/mL), and normetanephrine: 1200 pg/mL (normal 0-900 pg/mL)), confirming the diagnosis of pheochromocytoma [5]. Magnetic resonance imaging (MRI) and computed tomography (CT) imaging studies were performed to determine the location of the tumor. Figure 1 and Figure 2 demonstrate a 3 cm mass discovered in the left adrenal gland with evidence of local invasion into the surrounding tissues [6].

MRI of the brain was normal. Genetic testing for MEN and VHL was negative. The patient was started on an alpha-adrenergic blocker (phenoxybenzamine, 10 mg, p.o., 2-3 times daily, based on blood pressure) to prevent an intraoperative hypertensive crisis [1]. He underwent a laparoscopic left adrenalectomy following 10 d of phenoxybenzamine. Intraoperative findings confirmed the presence of left pheochromocytoma with local invasion into the adjacent tissues. The tumor was removed completely, and the postoperative period was uneventful. The patient was discharged on the third postoperative day with relatively normal blood pressure (120-135 systolic, 75-85 diastolic). Histopathological examination confirmed the diagnosis of pheochromocytoma. The tumor measured 3.5 cm in diameter and showed evidence of capsular and vascular invasion. The mitotic rate was low, and the Ki-67 index was 5%. The tumor was negative for malignancy markers. The patient was followed up in the clinic for six months postoperatively. He reported the resolution of his symptoms, and his blood pressure was normal on follow-up visits. Repeat imaging did not show any evidence of tumor recurrence.

## Discussion

Pheochromocytoma is a rare tumor that can present with a wide range of symptoms, making the diagnosis challenging. Biochemical testing for plasma catecholamines and metanephrines is the cornerstone of diagnosis [7]. Imaging studies like CT and MRI can locate the tumor and determine its extent of invasion. Alpha-blockers are used to control hypertension and prevent intraoperative hypertensive crises during surgical resection [6]. The prognosis of pheochromocytoma is generally good with surgical resection being the mainstay of treatment. However, the risk of recurrence and metastasis is higher in patients with malignant pheochromocytoma. Although the patient’s tumor was successfully resected and characterized, the continuing challenge following resection is to prevent the recurrence of the pheochromocytoma [8]. Measurement of plasma or urinary metanephrines and normetanephrine at 3-6 month intervals to monitor for biochemical evidence of recurrence during the first year and annually thereafter, with annual imaging is recommended. Given that a substantial proportion of pheochromocytomas are associated with hereditary syndromes, genetic counseling and possibly testing might be indicated, with specific follow-up guidelines depending on the underlying genetic alterations. By targeting the specific molecular pathways that drive tumor growth and evasion of the immune system, and by developing personalized treatment plans based on individual genetic profiles, medical practitioners may, in the future, allow improvement of clinical outcomes by preventing the recurrence of pheochromocytoma and other rare tumors.

Diagnostic challenge: Pheochromocytoma vs. hyperthyroidism or thyrotoxicosis

Distinguishing between pheochromocytoma and thyrotoxicosis can present a diagnostic challenge due to the overlapping multisystemic, nonspecific, and episodic symptomatology exhibited by both diseases. For example, both diseases can cause high blood pressure, leading to misdiagnosis if the blood pressure is solely relied upon. Other common features include tachycardia, palpitations, sweating, flushing, heat intolerance, anxiety, panic attacks, weight loss, and fatigue; thereby, making it difficult to pinpoint the underlying condition [9-11].

Recurrence following resection: Genes and pheochromocytoma

Approximately 40% of pheochromocytoma cases are known to be associated with germline mutations in one of the several susceptibility genes [12]. These genes include those involved in the Krebs cycle and electron transport chain, including SDHA, SDHB, SDHC, and SDHD, as well as VHL, RET, NF1, TMEM127, and MAX [13]. The identification of these genetic mutations has allowed for the early diagnosis and improved management of patients with hereditary pheochromocytoma. One of the most commonly mutated genes in pheochromocytoma is the succinate dehydrogenase (SDH) gene complex. Mutations in SDH genes can result in the accumulation of succinate, which may lead to the stabilization of hypoxia-inducible factor (HIF) and subsequent upregulation of genes involved in angiogenesis [13]. Other genes that have been implicated in the development of pheochromocytoma include the VHL tumor suppressor gene, RET (rearranged during transfection) proto-oncogene, neurofibromatosis type 1 (NF1), and the tumor suppressor gene transmembrane protein 127 (TMEM127) [14]. The identification of these genetic mutations has led to the development of genetic testing and screening programs for individuals at risk of developing pheochromocytoma. Individuals with a family history of pheochromocytoma or paraganglioma should undergo genetic testing to identify potential mutations [15]. Those with mutations in SDH genes or other predisposing genes should have surveillance imaging, on an annual basis, to detect potential tumors early [16].

Environment-gene interaction in pheochromocytoma

DNA methylation studies in pheochromocytoma have reported several differentially methylated genes that may be involved in tumorigenesis. The DNA methylation pattern of the promoter region of the *SDHC* gene was reported to be significantly different in pheochromocytoma compared to normal adrenal gland tissue, thereby suggesting a potential epigenetic mechanism for the development of this tumor [17]. The role of histone deacetylases (HDACs) in the development of pheochromocytoma appears to involve overexpression of HDAC1 and HDAC2. Inhibition of HDAC activity appears to decrease cell proliferation and induces cell death in pheochromocytoma cell lines [18]. The potential role of microRNAs (miRNAs) in the development of pheochromocytoma is suggested by reports that several miRNAs are dysregulated in pheochromocytoma. MiR-204 and miR-483-5p are reported to be downregulated in several other tumors and may, therefore, be responsible for tumor suppression in individuals without pheochromocytoma or other such tumors [19].

## Conclusions

This case report highlights the diagnostic and therapeutic challenges associated with pheochromocytoma, a rare tumor arising from the chromaffin cells in the adrenal medulla. The diagnosis of pheochromocytoma requires a high index of suspicion, given its multisystemic, nonspecific, and episodic symptoms. The biochemical testing for plasma catecholamines and metanephrines and imaging studies like CT and MRI play a crucial role in the diagnosis and management of this tumor. Surgical resection is the mainstay of treatment, with alpha-adrenergic blockers used to control systemic hypertension and prevent intraoperative hypertensive crises. The prognosis of pheochromocytoma is generally good with surgical resection, although the risk of recurrence and metastasis is higher in patients with malignant pheochromocytoma. Epigenetic mechanisms such as differential methylation of the DNA and the presence of micro-interfering RNAs suggest that environment-gene interaction plays a role in the susceptibility and recurrence of pheochromocytoma. This case report underscores the importance of early diagnosis, appropriate management, and follow-up for pheochromocytoma to ensure the best possible outcomes for patients.
